# Impact of switch from tenofovir disoproxil fumarate-based regimens to tenofovir alafenamide-based regimens on lipid profile, weight gain and cardiovascular risk score in people living with HIV

**DOI:** 10.1186/s12879-021-06479-9

**Published:** 2021-09-06

**Authors:** Pierre-Emmanuel Plum, Nathalie Maes, Anne-Sophie Sauvage, Frédéric Frippiat, Christelle Meuris, Françoise Uurlings, Marianne Lecomte, Philippe Léonard, Nicolas Paquot, Karine Fombellida, Dolores Vaira, Michel Moutschen, Gilles Darcis

**Affiliations:** 1 Infectious Diseases Department, Liège University Hospital, Liège, Belgium; 2 Biostatistics and Medico-Economic Information Department, Liège University Hospital, Liège, Belgium; 3 Diabetology Department, Liège University Hospital, Liège, Belgium; 4 AIDS Reference Laboratory, Liège University, Liège, Belgium

**Keywords:** Tenofovir alafenamide, Lipid, Weight gain, Cardiovascular risk, HIV

## Abstract

**Background:**

As cardiovascular diseases represent the main cause of non-AIDS related death in people living with HIV (PLWH) with undetectable viral load, we evaluated lipid profile, weight gain and calculated cardiovascular risk change after switching from tenofovir disoproxil fumarate (TDF)-based regimens to tenofovir alafenamide (TAF)-based regimens.

**Methods:**

For this retrospective study, we selected HIV-infected patients with suppressed viral load who fitted in one of the two groups below: First group (TDF/TDF): Patients treated continuously with TDF-based regimens. Second group (TDF/TAF): Patients treated with TDF-regimens during at least 6 months then switched to TAF-regimens while maintaining other drugs unchanged. Available data included date of birth, gender, ethnicity, lymphocyte T CD4^+^ count, weight, height, blood pressure, current/ex/non-smoker, diabetes mellitus, familial cardiovascular event, lipid profile, duration and nature of antiretroviral therapy. Lipid parameters, weight and calculated cardiovascular risk using 5-year reduced DAD score algorithm [Friis-Møller et al. in Eur J Cardiovasc Prev Rehabil 17:491–501, 2010] were analyzed in each groups.

**Results:**

Switching from TDF to TAF resulted in a significant increase in triglycerides levels, total cholesterol and HDL cholesterol. LDL cholesterol and total cholesterol/HDL ratio did not show significant changes. Calculated cardiovascular risk increased after switch from TDF- to TAF-based therapy.

**Conclusions:**

Together with favorable outcomes at the bone and kidney levels, potential negative impact of TAF on lipid profile should be included in the reflection to propose the most appropriate and tailored ARV treatment.

**Supplementary Information:**

The online version contains supplementary material available at 10.1186/s12879-021-06479-9.

## Introduction

Since the advent of antiretroviral therapy (ART), HIV has changed from a fatal disease to a chronic disease, with people living with HIV (PLWH) attaining a normal life expectancy [2–4].

However, despite efficient virological control, PLWH still experience excess morbidity and mortality compared to general population [4, 5]. Age-related illnesses such as cardiovascular disease, malignancies, osteoporosis and cognitive impairment are all more prevalent in the ageing population of PLWH. Some of them arise at an earlier age [4, 5]. In this regard, non-infectious co-morbidities have an growing effect on the prognosis of PLWH [6].

Cardiovascular diseases (CVD) represent one of the main causes of non-AIDS related death [6, 7]. It has emerged as a critical cause of death in PLWH relative to the diminishing incidence of opportunistic disease. CVD are estimated to account for 11–30% of mortality in PLWH [4, 8, 9]. The risk of myocardial infarction is almost doubled in PLWH [10].

PLWH with undetectable viral load under ART have an increased risk of CVD. It is thought to be due to several factors, including a persistent chronic atherogenic inflammatory state. The cause of this immune activation in treated HIV infection is unclear but may be related to various elements such as persistent low grade viral replication, direct effects of HIV proteins, viral co-infections (e.g. cytomegalovirus (CMV), hepatitis C virus), regulatory lymphocytes T cell (Treg) deficiency, decreased thymic function and increased gut microbial translocation [4, 11, 12].

Traditional risk factors for CVD are also common in PLWH: smoking, sedentary lifestyle, high blood pressure, high body mass index, hypercholesterolemia, diabetes, unhealthy diet and psychosocial stress are responsible for an increased CVD risk [13–15]. Also well-established is the association of lipid profile and cardiovascular morbidity and mortality in PLWH [1, 16]. The respective contributions of HIV infection itself, ART and traditional risk factors to the augmented CVD risk in PLWH are currently unknown.

Untreated PLWH with detectable viral load frequently develop dyslipidemia (elevated triglycerides levels and low levels of total cholesterol) due to HIV infection itself [17, 18]. ART, by reducing HIV viremia and inflammation, reduces the overall risk of CVD in PLWH [19]. However, some ARV medications may be associated with metabolic complications like dyslipidemia and increased CV events. For example, abacavir and some protease inhibitors (ritonavir, indinavir, lopinavir, darunavir) have been associated with an increased risk of CVD [4, 20]. Some ART like efavirenz, stavudine and zidovudine are associated with dyslipidemia [13]. In contrast, tenofovir reduces atherogenic lipids [21, 22] and heart failure risk [23]. This “lipid-friendly” effect is seen after switch from another nucleoside reverse transcriptase inhibitor (NRTI) to TDF-containing regimen [10].

The Data collection on Adverse Effects of Anti-HIV Drugs Study (DAD) equation was specifically developed to evaluate cardiovascular risk of HIV-infected patients. DAD equation takes into account, in addition to the traditional CVD risk factors, ART exposure. It is based on the results of a large multicenter cohort study conducted mainly in Europe and North America [1].

Studies demonstrated fat gain in ART-naïve PLWH due to restoration to health after initiation of ART. Otherwise, obesity and lipohypertrophy are over-represented in PLWH on ART. These fat storage alterations are of multifactorial origin: direct effect of HIV proteins and ARV on adipocytes, increased microbial translocation, tissue inflammation, genetic and other factors [24].

As efficacy of the latest generations of ART is generally high, their specific adverse event profile may guide when choosing the initial regimen and switching therapies [25].

Tenofovir alafenamide (TAF) has been introduced as a better prodrug of tenofovir. TAF achieves higher intracellular concentrations of tenofovir diphosphate (the active form of tenofovir) in mononuclear cells and hepatocytes but with lower plasma tenofovir concentration compare to its predecessor, the tenofovir disoproxil fumarate (TDF). These pharmacokinetic properties allow to reduce long term renal and bone toxicity compared to TDF [26, 27]. For this reason, TAF is often part of the recommended ART regimens in the current guidelines [28].

However, as TDF has a favorable effect on dyslipidemia by reducing atherogenic lipids [21], switching from TDF to TAF seems to worsen lipid profile [10, 29, 30] probably by several mechanisms including reduced levels of active serum form of tenofovir [22].

In this study, we aimed at figuring out the impact of switching from TDF to TAF on lipid profile, calculated cardiovascular risk score and weight gain.

## Methods

For this single center observational retrospective cohort study, we selected individuals from a database recording all HIV-infected outpatients attending CHU Liege hospital in Belgium on 18/01/19:

- First group (TDF/TDF): PLWH treated continuously with TDF-based regimens since 2016 or earlier with suppressed viral load for at least one year on 2016.
- Second group (TDF/TAF): PLWH treated with TDF-based regimens during at least six months then switched to TAF-regimens while maintaining other drugs unchanged (table S1.). TAF-regimens had to be continued during at least six months after switch. Viral load had to be suppressed for at least one year before switching.

Patients who modified their lipid lowering medications between available lipid measurements were excluded. Modification of other ARV drugs than TDF to TAF was also an exclusion criterion. Viral load had to be suppressed for at least one year before the first measurement of clinical and biological parameters in order to avoid modification of those parameters resulting from a 'return to health' effect.

Clinical and biological collected data included age, sex, weight, height, systolic blood pressure, current/ex/non-smoker, diabetes mellitus, total cholesterol (TC), HDL cholesterol, LDL cholesterol, TC/HDL cholesterol ratio, triglycerides, treated dyslipidemia, familial cardiovascular event, lymphocyte T CD4^+^ level, duration and type of previous and current ART and concomitant treatment related to comorbidities.

For lipid parameters in TDF/TDF group, we selected last lipid level values available in 2016 and 2018 with a minimum exposure of 6 months to TDF for the first value selected. For lipid parameters in TDF/TAF group, we selected lipid level values at baseline with a minimum exposure of 6 months to TDF and at 6 months minimum after switch.

Cardiovascular risk score was calculated using reduced DAD (DAD-R) model equation from Friis-Møller et al. appendix [1]. Furthermore, it can be calculated on Risk Assessment Tool System of “CHIP-Centre of Excellence for Health, Immunity and Infections” website. Lipid profiles and calculated cardiovascular risks were analyzed and compared in each groups.

All data were reviewed before being analyzed. Characteristics of the analyzed cohort are presented in Table 1.

**Table. 1:** Characteristics of TDF/TDF (N = 31) and TDF/TAF groups (N = 98)

	TDF/TDF groupN = 31	TDF/TAF groupN = 98	Comparisonp-value
Age (years)	50 ± 11	50 ± 12	0.79
Sex = Male	22 (71.0)	63 (64.3)	0.49
Smoking			0.26
Non-smoker	16 (51.6)	51 (53.7)	
Ex-smoker	3 (9.7)	19 (20.0)	
Smoker	12 (38.7)	25 (26.3)	
Total	*31 (100.0)*	*95 (100.0)*	
Ethnicity			0.41
Caucasian	15 (48.4)	54 (55.1)	
African	16 (51.6)	41 (41.8)	
Other	0 (0.0)	3 (3.1)	
Treated hypertension	5 (16.1)	25 (25.5)	0.28
Treated dyslipidemia	4 (12.9)	11 (11.2)	0.80
Familial cardiovascular event	1 (3.2)	4 (4.1)	0.83
Diabetes	1 (3.2)	9 (9.2)	0.28
	In 2016	Before switch	
Triglycerides (mg/dL)	134 ± 82	123 ± 65	0.43
Total cholesterol (mg/dL)	184 ± 39	183 ± 38	0.90
LDL cholesterol (mg/dL)	107 ± 34	103 ± 32 (N = 97)	0.71
HDL cholesterol (mg/dL)	51 ± 12	56 ± 17 (N = 97)	0.18
Non-HDL cholesterol (mg/dL)	133 ± 39	128 ± 37 (N = 97)	0.45
Total cholesterol /HDL ratio	3.8 ± 1.2	3.6 ± 1.2 (N = 97)	0.30
Weight (kg)	74.7 ± 14.8	77.8 ± 15.9	0.34
BMI (kg/m^2^)	25.1 ± 4.8	26.3 ± 4.9 (N = 88)	0.24
DAD-R (%)	2.1 (0.9; 3.2) (N = 30)	1.5 (0.6; 4.0) (N = 76)	0.50
CD4 (10^3^/mm^3^)	712 (509; 903)	683 (497; 928) (N = 97)	0.97
Time on TDF (years)	6 (5; 8)	6 (2; 8)	0.11

The Ethical Committee (EC) of the University Hospital of Liège (full name: Comité d'éthique Hospitalo-Facultaire Universitaire de Liège), Belgium, approved the study protocol. Patients were informed of data collection by their treating physician and patients could object to further collection of clinical data according to an opt-out procedure. All patients included were assigned unique identification numbers to anonymize the data and protect confidentiality. All methods were carried out in accordance with relevant guidelines and regulations. Informed consent was not required for this study, according to the EC (Comité d'Éthique Hospitalo-Facultaire Universitaire de Liège) recommendations.

### Statistical analyses

Data were summarized as mean and standard deviation (SD) or median and interquartile range (P25–P75) for quantitative variables while frequency tables were used for the categorical findings. Evolution of lipid parameters and weight was investigated by Student t test for paired samples. Since the distribution of DAD-R and CD4 lymphocyte count is not symmetric, nonparametric tests were used to analyze those variables (Kruskal Wallis and signed rank for paired samples tests). Longitudinal mixed effect multiple regression modeling approach with a change point at TAF initiation and including potential confounding variables were used to assess TAF effect. Variables with univariate significant impact (at p < 0.10 level) or clinically identified as potential confounder were included in the multivariate models. A log-transformation was applied for DAD-R modelling, and since those variables were directly or indirectly part of the score calculation, covariates were not included in the model. Missing data were not replaced and data analysis was carried out using SAS (version 9.4 for Windows). Results were considered significant at the 5% critical level (p < 0.05).

## Results

The TDF/TDF group included 31 patients with a TDF treatment duration ranging from 2 to 10 years (Table 1). The TDF/TAF group included 98 patients with TDF treatment duration, before switching, ranging from 6 months to 13 years and TAF treatment duration ranging from 9 months to 2.5 years at the time of data collection. ARV drug regimens in TDF/TAF group before and after switch are summarized in Additional file 1: Table S1.

TDF/TDF and TDF/TAF groups can be considered homogeneous for clinical and biological characteristics (Table 1). Median age was 50 years old in both groups. Most of the participants were male in each group. A short majority of the participants were non-smokers. Groups were also comparable regarding lipid profile, weight and BMI (Table 1).

In the TDF/TDF group, total cholesterol decreased significantly between 2016 and 2018 (p = 0.028) (Table 2A). Triglycerides (p = 0.26), HDL cholesterol (p = 0.44), LDL cholesterol (p = 0.38), non-HDL cholesterol (p = 0.089) and total cholesterol/HDL ratio (p = 0.53) did not significantly change (Table 2A.). In the TDF/TAF group, triglycerides (p = 0.0026), total cholesterol (p = 0.0026), HDL cholesterol (p = 0.0084) and non-HDL cholesterol (p = 0.049) increased significantly post-switch. LDL cholesterol (p = 0.48) and total cholesterol/HDL cholesterol ratio (p = 0.77) did not change (Table 2B.). Impact of TAF treatment on triglycerides (p = 0.034), total cholesterol (0.042), and HDL cholesterol (p = 0.0059) were significant (Additional file 1: Table S2).

**Table. 2:** Evolution of lipid parameters

A. TDF/TDF group, Mean [95%CI] (N = 31)					
	N	2016	2018	Evolution 2018–2016	p-value
Triglycerides (mg/dL)	31	134 [104;164]	116 [91;141]	− 18 [− 49;13]	0.26
TC (mg/dL)	31	184 [170;199]	176 [161;191]	− 8.5 [− 16;− 1.0]	**0.028**
LDL Cholesterol (mg/dL)	1	107 [94;119]	103 [89;117]	− 3.8 [− 12;4.8]	0.38
HDL Cholesterol (mg/dL)	31	51 [47;56]	50 [45;55]	− 1.2 [− 4.3;1.9]	0.44
TC/HDL ratio	31	3.8 [3.4;4.3]	3.7 [3.3;4.2]	− 0.087 [− 0.37;0.19]	0.53

B. Evolution of lipid parameters in TDF/TAF group, Mean [95%CI] (N = 98)					
	N	Before (on TDF)	After (on TAF)	Evolution After**–**Before	p-value
Triglycerides (mg/dL)	98	123 [110;136]	143 [124;161]	20 [7;33]	**0.0026**
TC (mg/dL)	98	183 [176;191]	192 [185;199]	8.7 [3.1; 14]	**0.0026**
LDL Cholesterol (mg/dL)	95	103 [97;110	105 [99;111]	1.7 [− 3.0;6.4]	0.48
HDL Cholesterol (mg/dL)	96	56 [52;53]	59 [55;63]	2.9 [0.75;5.0]	**0.0084**
TC/HDL ratio	96	3.6 [3.3;3.8]	3.5 [3.3;3.8]	− 0.023 [− 0.18;0.13]	0.77

Weight, and so Body Mass Index (BMI), evolution showed a non-significant decrease in the TDF/TDF group (p = 0.38) (Table 3A), a non-significant increase in the TDF/TAF group (p = 0.21) (Table 3B.), and a global trend in weight gain after switch to TAF (p = 0.058) (Table S2) in the linear mixed regression models.

**Table. 3:** Weight evolution

A. TDF/TDF group, Mean [95% CI]					
	N	2016	2018	Evolution 2018–2016	p-value
Weight (kg)	30	74.9 [69.3;80.5]	74.3 [68.2;80.4]	− 0.63 [− 2.1;0.81]	0.38

B. TDF/TAF group, Mean [95%CI]					
	N	Before (on TDF)	After (on TAF)	Evolution After–Before	p-value
Weight (kg)	83	78.7 [75.2;82.2]	79.6 [76.3;82.9]	0.94 [− 0.55;2.4]	0.21

Cardiovascular (CV) risk did not change in TDF/TDF group between 2016 and 2018 (p = 0.32) (Table 4A.) but increased when patients switched from TDF to TAF based regimens (p = 0.0008) (Table 4B.). DAD-R risk score increased from 1.6% (95% CI 1.2 to 2.1) under TDF to 1.8% (95% CI 1.4 to 2.4) under TAF (total increase of 12.5% after the switch). Nevertheless, the impact of TAF treatment was not confirmed by the modelling approach with a change point at TAF initiation (p = 0.75) (Table S2.).

**Table. 4:** Evolution of cardiovascular risk

A. TDF/TDF group, Mean [95% CI]					
	N	2016	2018	Evolution 2018–2016	p-value
DAD-R	26	1.8 [1.3;2.7]^(a)^	1.9 [1.3;2.7]^(a)^	0.20 [− 0.19;0.59]	0.32

**B.** TDF/TAF group, Mean [95%CI]					
	N	Before (on TDF)	After (on TAF)	Evolution After–Before	p-value
DAD-R	64	1.6 [1.2;2.1]^(a)^	1.8[1.4;2.4]^(a)^	0.43 [0.090;0.76]	**0.0008**

^(a)^ Given the right skew of DAD-R, mean and 95%IC were calculated using log transformation. The results were back-transformed to the original scale

## Discussion

Our study aimed at analyzing the impact of switching from TDF-based regimen to a TAF-based regimen on lipid profile, CV risk score and weight gain in virally suppressed individuals. As already demonstrated by other research groups [10, 27, 29–31], we confirmed a worsening of lipid profile after switching from TDF- to TAF-based ARV regimens. Indeed, we observed significant increase in triglycerides, total cholesterol levels and in non-HDL cholesterol, often considered as the best measure of cholesterol levels carried by all proatherogenic lipoproteins, post-switch. However, contrary to some of these studies, we showed no significant evolution of LDL cholesterol and total cholesterol/HDL ratio.

These elements are significant as dyslipidemia is already particularly common in PLWH on ART of any kind. Interestingly, a recent study showed that changes in lipid profile are reversible after switching back to TDF [30]. According to some authors, statins prior to the switch seem to protect from the increases in lipids [10].

We further showed the evidence of an increase in calculated CVD risk according to reduced DAD algorithm when switching from TDF to TAF due to increases in total cholesterol and triglycerides levels, although this effect was not confirmed in the linear mixed regression models. The impact of TAF on CVD risk should thus be investigated on larger cohort studies.

This observation is important as PLWH are more susceptible to develop CVD even if viral load is undetectable under ART [29], making of CVD a major cause of non-AIDS morbidity and mortality in PLWH. Although the relative impact of ARV drugs compared to traditional risk factors and HIV infection itself is unknown, it should be taken into account while considering the various treatment options, especially in patients who are at risk of developing CVD. Nevertheless, prospective controlled studies are needed to assess the effect of the switch on cardiovascular outcomes.

Weight gain following the initiation of ART has been reported across multiple settings and study populations [31]. Part of it may simply reflect a return to health among those who are underweight at the time of ART initiation. This is particularly evident in individuals with low baseline CD4^+^ T cell count and high baseline HIV RNA levels [32]. A potential impact of specific ARV drugs has also recently been highlighted, with some specific populations being at higher risk of gaining weight [33–36]. In those studies, dolutegravir (DTG) was associated with excess weight gain as compared to efavirenz (EFV), particularly when associated with TAF–emtricitabine. It should be noted that the clinical relevance of this weight gain is still unclear. Switch studies avoiding confounding by return to health phenomenon are needed to confirm conclusions regarding the ARV impact on weight gain. In our study, we observed a trend to a small weight gain (+ 0.94 kg) in the switch group but it did not reach statistical relevance. In the linear mixed regression models, we observed a trend towards weight increase (p 0.058) following switch to TAF. This important observation is in agreement with recent findings of the ADVANCE trial showing that weight increase (both lean and fat mass) was greatest in the TAF-based group [30].

The main limitations of our study are its retrospective nature and the low number of patients included in the TDF/TDF group. However, this number is due strict inclusion/exclusion criteria needed to perform a relevant comparison of both groups. Indeed, the strengths of our study is that all included PLWH were effectively virally-suppressed to avoid a 'return to health' effect. All participants maintained their ARV regimens unchanged (except for TDF in TDF/TAF group) and did not modify their lipid-lowering therapy to avoid impact of other medications. Another quality of our study is its “real-world” setting and data with older patients compared to generally younger patients enrolled in clinical trials. Moreover, we used non-HDL cholesterol that is considered the best measure of the cholesterol carried by all proatherogenic lipoproteins.

In conclusion, TAF is a new interesting drug with a security profile which is attractive as far as renal and bone complications are concerned, due to a much lower serum concentration and a higher intracellular concentration of tenofovir diphosphate (active form of tenofovir). Nevertheless, switching from TDF to TAF leads to a significant increase in atherogenic lipid levels and to a higher risk of requiring lipid-lowering medication [27]. We further demonstrated an increased cardiovascular risk score post-switch. Thus, the relative impact of lipid profile worsening effect of switching from TDF-based regimens to TAF-based regimens should be taken into account alongside renal and bone safety especially in patients with dyslipidemia and/or high cardiovascular risk. Further larger studies with long term follow-up are needed to know if cardiovascular event incidence corresponds to predicted cardiovascular risk using reduced DAD algorithm. Finally, we showed that switch to TAF-based regimen is associated with a significant weight gain in virally-suppressed females, a phenomenon of unknown clinical impact but which undoubtedly deserves to be further studied.

## Supplementary Information

**Additional file 1: Table S1**. TAF-based regimens after switch from TDF-Based regimens.** Table S2**. Evolution of lipid parameters, weight and cardiovascular risk - Linear Mixed Regression Models.

## Data Availability

The datasets used and/or analysed during the current study are available from the corresponding author on reasonable request.
