# Supplementary material for: Impact of switch from tenofovir disoproxil fumarate-based regimens to tenofovir alafenamide-based regimens on lipid profile, weight gain and cardiovascular risk score in people living with HIV
Source: BMC Infect Dis. 2021 Sep 6;21:910. doi: 10.1186/s12879-021-06479-9 (PMC8420041; doi:10.1186/s12879-021-06479-9)
Supplement: Supplementary file 1 — Additional file 1: Table S1. TAF-based regimens after switch from TDF-Based regimens. Table S2. Evolution of lipid parameters, weight and cardiovascular risk - Linear Mixed Regression Models. [file 12879_2021_6479_MOESM1_ESM.docx]

**Impact of switch from tenofovir disoproxil fumarate-based regimens to tenofovir alafenamide-based regimens on lipid profile, weight gain and cardiovascular risk score in people living with HIV**

**Authors:** Pierre-Emmanuel Plum^1^, Nathalie Maes^2^, Anne-Sophie Sauvage^1^, Frédéric Frippiat^1^, Christelle Meuris^1^, Françoise Uurlings^1^, Marianne Lecomte^1^, Philippe Léonard^1^, Nicolas Paquot^3^, Karine Fombellida^1^, Dolores Vaira^4^, Michel Moutschen^1,4^ and Gilles Darcis^1*^

* Corresponding author: [gdarcis@chuliege.be](mailto:gdarcis@chuliege.be) ; +3243667235

Affiliations:

1 Infectious Diseases department, Liège University Hospital, Belgium

2 Departments of Biostatistics and Medico-Economic Information, Liège University Hospital, Belgium

3 Diabetology department, Liège University Hospital, Belgium

4 AIDS Reference Laboratory, Liège University, Belgium

**Supplementary data**

| **Table S1.** **TAF based regimens after switch** | | |
| --- | --- | --- |
| TDF | TAF | N (%) |
| Stribild | Genvoya | 55 (56.1) |
| Eviplera | Odefsey | 15 (15.3) |
| Truvada-Viramune ou Resolsta-Truvada | Descovy-Viramune | 14 (14.3) |
| Tivicay-Truvada | Descovy-Tivicay | 8 (8.2) |
| Isentress-Truvada | Descovy-Isentress | 4 (4.1) |
| Norvir-Prezista-Truvada | Descovy-Norvir-Prezista | 1 (1.0) |
| Norvir-Reyataz-Truvada | Descovy-Norvir-Reyataz | 1 (1.0) |
|  |  |  |
| *Total* |  | *98 (100.0)* |

| **S2.** Evolution of lipid parameters, weight and cardiovascular risk - Linear Mixed Regression Models (N=126 patients, 252 measurements) | | | |
| --- | --- | --- | --- |
|  |  | Coef. ± SE | p-value |
| Triglycerides (mg/dL) | Intercept | 110 ± 23 | - |
|  | Age (years) | 0.56 ± 0.46 | 0.22 |
|  | Sex (ref=Male) | -36 ± 11 | **0.0008** |
|  | Ethnicity : Caucasian (=ref) vs African | -18 ± 11 | 0.096 |
|  | Time on TDF at inclusion (years) | 0.92 ± 1.6 | 0.55 |
|  | Treatment : TDF (=ref) vs TAF | 21 ± 9.8 | **0.034** |
|  |  |  |  |
| TC (mg/dL) | Intercept | 163 ± 12 | - |
|  | Age (years) | 0.26 ± 0.23 | 0.25 |
|  | Sex (ref=Male) | 10 ± 5.4 | 0.057 |
|  | Ethnicity : Caucasian (=ref) vs African | 0.11 ± 5.3 | 0.98 |
|  | Time on TDF at inclusion (years) | 0.23 ± 0.78 | 0.77 |
|  | Treatment : TDF (=ref) vs TAF | 10 ± 4.9 | **0.042** |
|  |  |  |  |
| LDL Cholesterol (mg/dL) | Intercept | 101 ± 10 | - |
|  | Age (years) | 0.051 ± 0.20 | 0.79 |
|  | Sex (ref=Male) | 3.7 ± 4.6 | 0.43 |
|  | Ethnicity : Caucasian (=ref) vs African | -0.86 ± 4.6 | 0.85 |
|  | Time on TDF at inclusion (years) | -0.31 ± 0.68 | 0.65 |
|  | Treatment : TDF (=ref) vs TAF | 0.35 ± 4.2 | 0.93 |
|  |  |  |  |
| HDL Cholesterol (mg/dL) | Intercept | 41 ± 4.6 | - |
|  | Age (years) | 0.091 ± 0.091 | 0.32 |
|  | Sex (ref=Male) | 14 ± 2.1 | **<0.0001** |
|  | Ethnicity : Caucasian (=ref) vs African | 4.0 ± 2.1 | 0.063 |
|  | Time on TDF at inclusion (years) | 0.29 ± 0.31 | 0.36 |
|  | Treatment : TDF (=ref) vs TAF | 5.4 ± 2.0 | **0.0059** |
|  |  |  |  |
| TC/HDL ratio | Intercept | 4.2 ± 0.35 | - |
|  | Age (years) | -0.001 ± 0.007 | 0.87 |
|  | Sex (ref=Male) | -0.72 ± 0.16 | **<0.0001** |
|  | Ethnicity : Caucasian (=ref) vs African | -0.29 ± 0.16 | 0.072 |
|  | Time on TDF at inclusion (years) | -0.020 ± 0.024 | 0.39 |
|  | Treatment : TDF (=ref) vs TAF | -0.12 ± 0.15 | 0.44 |
|  |  |  |  |
| Weight (kg) | Intercept | 77 ± 4.5 | - |
|  | Age (years) | -0.020 ± 0.087 | 0.81 |
|  | Sex (ref=Male) | -15 ± 2.1 | **<0.0001** |
|  | Ethnicity : Caucasian (=ref) vs African | 6.5 ± 2.1 | **0.0018** |
|  | Time on TDF at inclusion (years) | 0.44 ± 0.30 | 0.14 |
|  | Treatment : TDF (=ref) vs TAF | 3.6 ± 1.9 | 0.058 |
|  |  |  |  |
| DAD-R | Intercept | 0.53 ± 0.089 | - |
|  | Treatment : TDF (=ref) vs TAF | 0.047 ± 0.15 | 0.75 |
